# RBP7 knockdown inhibits proliferation of human hepatocellular carcinoma and activates the p38 MAPK pathway

**DOI:** 10.3389/fonc.2025.1592616

**Published:** 2025-06-25

**Authors:** Jinhai Li, Huawei Zhai, Fujing Cai, Xian Zhang, Yu Zhou, Shuqun Li, Huachun Song, Haifeng Zhang, Guangzheng Sun, Minghui Zhu, Jing Yuan, Ningxin Zhang, Maolin Yan

**Affiliations:** ^1^ Shengli Clinical Medical College of Fujian Medical University, Fuzhou, Fujian, China; ^2^ Department of Hepatobiliary Pancreatic Surgery, The Third Affiliated Hospital of Wenzhou Medical University, Wenzhou, Zhejiang, China; ^3^ Department of Infectious Diseases, The Third Affiliated Hospital of Wenzhou Medical University, Wenzhou, Zhejiang, China; ^4^ Zhejiang Provincial Key Laboratory of Medical Genetics, Key Laboratory of Laboratory Medicine, Ministry of Education, Wenzhou, China; ^5^ School of Laboratory Medicine and Life Science, Wenzhou Medical University, Wenzhou, Zhejiang, China; ^6^ Department of Hepatobiliary and Pancreatic Surgery, Affiliated Hospital of Guilin Medical University, Guilin, Guangxi, China; ^7^ Department of Medicine Radiology, The Zhejiang Hospital of Integrated Traditional Chinese and Western, Hangzhou, Zhejiang, China; ^8^ Department of Hepatobiliary Pancreatic Surgery, Fujian Provincial Hospital, Fuzhou, Fujian, China

**Keywords:** hepatocellular carcinoma, p38, MAPK, RBP7, retinoid, cell proliferation

## Abstract

**Background:**

Retinoid metabolism is critical for maintaining liver homeostasis, and its dysregulation is closely associated with liver diseases. Retinol binding protein 7 (RBP7) involves in retinoids transport, particularly in liver, indicating its importance in hepatic functions. However, its specific role in hepatocellular carcinoma (HCC) tumorigenesis remains unclear and needs to further investigation.

**Methods:**

Bioinformatics was employed to assess RBP7 expression across different cohorts. The expression level of RBP7 in cells were further validated using qRT-PCR and western blot. Additionally, we investigated the impact of RBP7 knockdown on cell cycle-related genes, apoptosis-related proteins, and p38 MAPK signaling activity. Functional assays, including CCK8, colony formation, flow cytometry (FACS) analysis, Annexin V/7-AAD staining and xenograft tumor assay, were performed to determine the *vitro* and *in vivo* role of RBP7. Survival analysis was conducted to evaluate the correlation between RBP7 expression and the prognosis of HCC patients.

**Results:**

RBP7 is frequently elevated in HCC tumor tissues, particularly in early-stage patients. Notably, high RBP7 expression is closely correlated with overall survival (OS) and disease-specific survival (DSS) in HCC patients. Knockdown of RBP7 inhibited cell proliferation *in vitro* and suppressed tumor growth *in vivo* by inducing cell cycle arrest and apoptosis. Mechanistically, we found that RBP7 knockdown-induced suppression of HCC cell proliferation was associated with increased phosphorylation of p38 MAPK.

**Conclusion:**

Our findings demonstrate that RBP7 suppression activates p38 MAPK signaling pathway, leading to impaired cell proliferation. These results suggest that RBP7 may serve as both a prognostic biomarker and a promising therapeutic target for HCC.

## Introduction

Hepatocellular carcinoma (HCC) is the predominant histologic type of primary liver cancer, accounting for approximately 75% of all liver cancer cases (1). Despite significant advances in diagnosis and therapeutic approaches, the prognosis for HCC patients remains poor due to high rates of recurrence and metastasis (2, 3). Thus, a deeper understanding of the molecular mechanisms driving HCC progression is essential to optimize the therapeutic strategies and improve patient outcomes.

Retinoids, encompassing all metabolic intermediates and chemicals with structures similar to vitamin A (retinoic acid, RA), exerts a pivotal role in modulating essential physiological processes such as cell proliferation, differentiation, apoptosis, vision, embryonic development, and immunomodulation (4, 5). The liver is the primary site for retinoids storage and metabolism, with hepatic stellate cells (HSCs) storing retinoids predominantly as retinol (6). It is well known that the effects of retinoids are mediated by a network of specialized retinoids-interacting proteins, including cellular retinoid-binding proteins (CRBPs), retinoid X receptors (RXRs), retinoic acid receptors (RARs), and retinol-binding proteins (RBPs). These proteins regulate retinoid metabolism, transport, and signaling (7). Among them, RBPs serve as the principal transporters, facilitating retinoids transport from the liver to peripheral tissues. Dysregulation or dysfunction of RBPs has been closely linked to a variety of human diseases, including type 2 diabetes, cardiovascular disorders, and cancers (7, 8).

RBP7, a member of RBP family, exhibits binding affinity for 13-cis-retinol, all-transretinal, and 9-cis-retinol, but not retinoic acids (9). RBP7 act as a transcriptional target of peroxisome proliferator-activated receptor gamma (PPARγ) and contributes to PPARγ-mediated antioxidant response via a feedback loop (10, 11). Additionally, RBP7 also plays an important role in adipogenesis, with *RBP7* knockout mice displaying reduced adiposity and increased lean body mass (11). Aberrant expression of RBP7 has been implicated in several cancers, including breast cancer, bladder urothelial carcinoma and colon cancer, where its expression closely correlates with patients prognosis (12–15). The loss of RBP7 expression has been shown to promote tumor progression by affecting the PPAR and PI3K/AKT signaling pathways in estrogen receptor-positive (ER+) breast cancer (12). It has also been proposed that RBP7 may serve as a prognostic biomarker in colon cancer and functionally contributes to the malignant phenotype by promoting epithelial-mesenchymal transition (EMT) and tumor invasion (13). However, the functional significance of RBP7 in HCC remains elusive.

Here, we investigated the expression pattern of RBP7 in HCC and characterized its functional role in tumorigenesis.

## Materials and methods

### Patients and specimens

A cohort of 53 patients diagnosed with primary HCC was recruited from the Third Affiliated Hospital of Wenzhou Medical University (Zhejiang, China). Paired tumor tissues and paracarcinoma tissues were collected and used for assessing the expression of RBP7 by qRT-PCR. Informed consent was obtained from all participants prior to sample collection. The study was approved by the Research Ethics Committee of the Third Affiliated Hospital of Wenzhou Medical University (YJ2022006).

### Cell culture and transfection

The resources and cultural procedure of cell lines were depicted (16). All cell lines were characterized by DNA fingerprinting and isozyme detection.

Nonsense control siRNA (siNC: 5’-UUCUCCGAACGUGUCACGUUU-3’), RBP7 siRNAs (siRBP7-1: 5’- GCCUAAGGAACUACUUUGUUU-3’; siRBP7-2: 5’-GUUUGGUUAUCUGGGACAAUU-3’) and p38 siRNA (sip38: 5’-UUGGUAGAUAAGGAACUGAAC-3’) were synthesized by GenePharma (Shanghai, China) and cells transfection were conducted with Lipofectamine 3000 (Life Technologies Inc., Massachusetts, USA) according to the manufacturer’s protocols.

### RNA extraction and quantitative real-time PCR

Total RNA extraction and qRT-PCR was performed as previously depicted (17). The primer sequences for qRT-PCR were shown as follows: 5’-GCAGCGACAACTTCGAGGGCTA-3’ (forward) and 5’-CACGAGTCCTCGTCGCCAAATG-3’ (reverse) for *RBP7*, 5’-AGGGCTTCCTGGACACG-3’ (forward) and 5’-GCATGGTTACTGCCTCTGG-3’ (reverse) for *p16*, 5’-GCCCGTGAGCGATGGAA-3’ (forward) and 5’-CGAGGCACAAGGGTACAAGAC-3’ (reverse) for *p21*, 5’-AGAGGGCAAGTACGAGTGG-3’ (forward) and 5’-CAGGTCGCTTCCTTATTCC-3’ (reverse) for *p27*, 5’-AAGCGGTCCCGTGGATAGA-3’ (forward) and 5’-TCCGGTATTCGCAGAAGTCC-3’ (reverse) for *Bcl-2*, 5’-GGAGCTGGTGGTTGACTTTCT-3’ (forward) and 5’-CCGGAAGAGTTCATTCACTAC-3’ (reverse) for *Bcl-xL*, 5’-ACCCTAGAGACATGGAGAAG-3’ (forward) and 5’-AGCTATCTTCCAGCCTGTCT-3’ (reverse) for *BID*, and 5’-TGCGTTACACCCTTTCTTGACA-3’ (forward) and 5’-GCAAGGGACTTCCTGTAACAATG-3’ (reverse) for *β-actin*. 2^-ΔΔCt^ was used to calculate relative mRNA levels and *β-actin* was used as internal control.

### Western blot

Cells were collected and lysed for western blot as previously described (18). The antibodies were shown as follows: rabbit α-RBP7 (A15939, ABclonal, Woburn, USA), mouse α-cyclin D1 (60186-1-lg, Proteintech, Rosemont, USA), rabbit α-cyclin A2 (18202-1-AP, Proteintech), rabbit α-PARP1 (66520-1-lg, Proteintech), rabbit α-caspase 9 (#9661, CST), rabbit α-cleaved caspase 7 (#9661, CST), rabbit α-cleaved caspase 3 (#9661, CST), rabbit α-p38 (#8690, CST), rabbit α-p-p38 (#4511, CST, Boston, USA) and secondary antibodies (S0001 and S0002, Affinity Biosciences, Changzhou, China).

### Cell proliferation, cell cycle and apoptosis analysis

Cell proliferation was examined as previously depicted (19). Briefly, for CCK8 assay, siRNA-transfected cells were seeded in 96-well plates at a density of 1500 cells per well, and proliferation was measured using CCK8 kit after culture for indicated period. For colony formation assay, 500 siRNA-transfected cells were plated in 6-well plate, and colonies were stained with 0.1% crystal violet after culture for 10 days and counted manually. The effects of RBP7 knockdown on cell cycle and apoptosis were assessed as described previously (17).

### Subcutaneous xenograft experiments

Animal experiments were approved by the Ethics Committee for Laboratory Animals of the Wenzhou Medical University and performed as previously described (20). Briefly, 3–4-weeks-old female BALB/c nude mice were randomly divided into three groups (n=5 per group).A total of 5×10^6^ Huh7 cells stable RBP7 knockdown or control cells were subcutaneously injected into the right axillary fossa of each mouse. Tumors dimensions were measured every three days using a caliper, and tumor volume (V) was calculated using the formula V= (length × width^2^)/2.

### Statistical analysis

Statistical analyses were performed using Graphpad Prism 7. Kaplan–Meier method and logrank test were used to estimate the probability of differences in overall survival (OS) and disease-specific survival (DSS). The Pearson’s *χ* ^2^ test was used to evaluate the relationship between RBP7 expression and clinicopathological parameters. Students’ two-tailed *t* test was used to assess the significance of different groups and *P<*0.05 was considered statistically significant.

## Results

### The expression of RBP7 is frequently elevated in HCC patients

**Figure 1:**
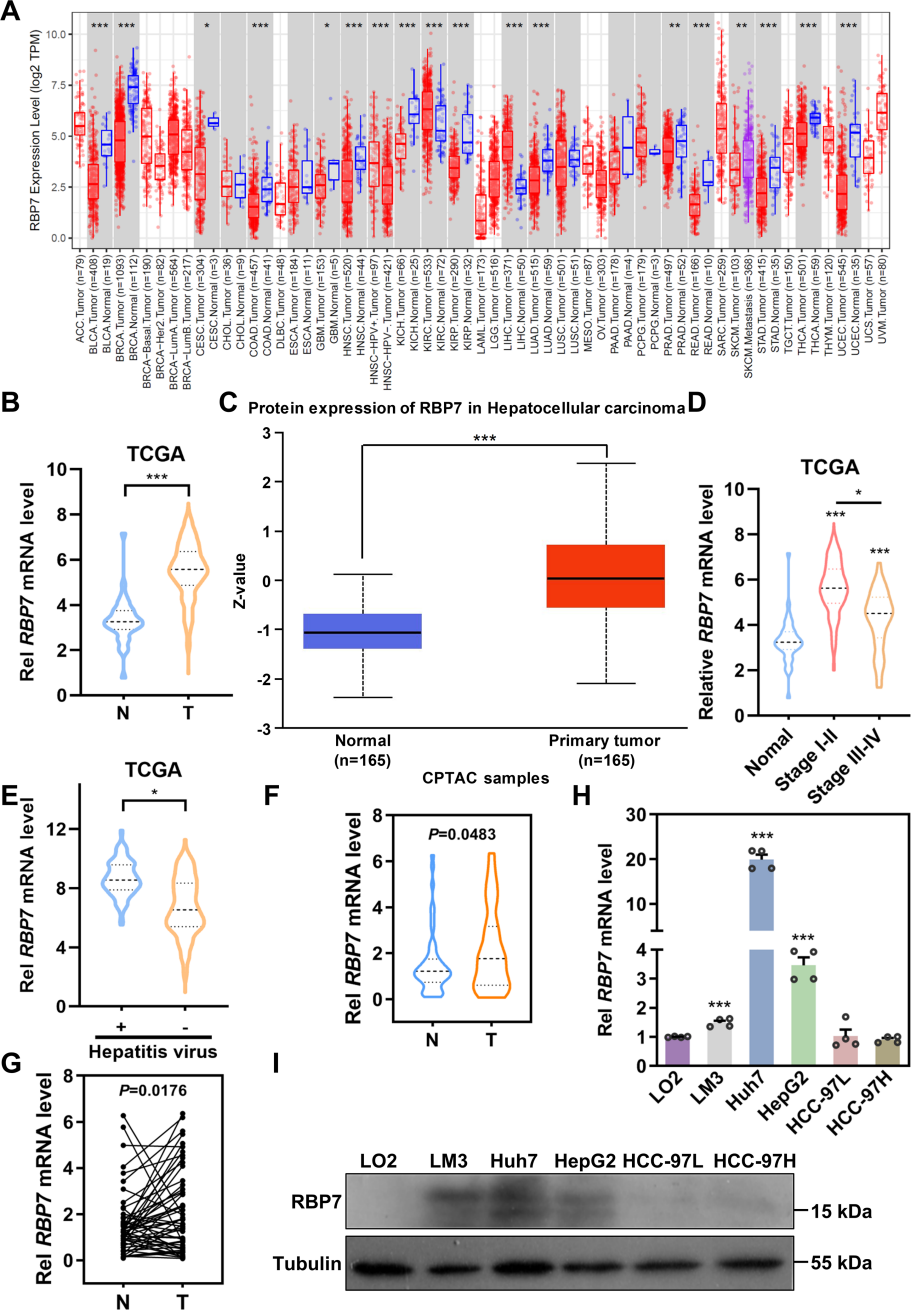
RBP7 is frequently elevated in HCC tissues and cells. **(A)** Expression of *RBP7* mRNA across different types of cancer based on TCGA cohorts. Expression of RBP7 in HCC was analyzed using TCGA **(B)** and CPTAC **(C)** cohorts. Expression of *RBP7* mRNA in different stages of HCC patients **(D)** or patients with or without hepatitis virus infection **(E)**. **(F)** *RBP7* mRNA level assessed by qRT-PCR in tumor (T) and paired adjacent non-cancerous (N) tissues from 53 HCC patients. **(G)** Expression of *RBP7* mRNA in 53 cases of HCC patients was calculated. qRT-PCR **(H)** or western blot **(I)** results showing RBP7 expression in normal hepatocytes LO2 cells and HCC cells. All experiments were performed at least three times. Data are presented as mean ± SD. *P* values were calculated using Student’s *t* test, *P< 0.05, **P< 0.01 and ***P< 0.001.

Previous studies have identified chromosome 1p36.22 as a susceptibility locus for HBV-related HCC, while the region is also amplified in primary gastric adenocarcinomas (21, 22), further implicating genes within this locus in tumorigenesis across cancer types. Through systematic screening of this locus, we identified *RBP7* as a candidate gene. Analysis of TCGA data revealed that *RBP7* undergoes frequent genomic alterations across multiple cancer types, with amplification occurring in 0.54% and deletion in 1.34% of HCC cases (**Supplementary Figure S1A**). To further characterize its potential role in HCC pathogenesis, we performed comprehensive expression analyses using both TCGA and CPTAC datasets through online bioinformatics platforms (http://ualcan.path.uab.edu/index.html). The results showed a consistent upregulation of RBP7 in HCC at both mRNA and protein levels (*P <*0.0001, **Figures 1A–C**). Stage-specific analysis showed particularly pronounced RBP7 expression in early-stage HCC patients relative to advanced-stage cases (**Figure 1D**), though no significant differences was observed between N0 and N1 patients (**Supplementary Figure S1B**). Notably, hepatitis virus-positive HCC patients exhibited higher RBP7 levels than their virus-negative counterparts (**Figure 1E**). This finding was further validated in our clinical cohort of 53 primary HCC cases, where tumor tissues showed significantly increased *RBP7* mRNA expression compared to matched normal tissues (*P* < 0.05, **Figure 1F**), with 62.3% (33/53) of cases demonstrating tumor-specific RBP7 upregulation (**Figure 1G**). Clinicopathological correlation analysis identified a positive correlation with patient age and a negative association with poor tumor differentiation (*P*<0.05, **Table 1**). At the cellular level, RBP7 expression was markedly elevated in LM3, Huh7, and HepG2 cell lines compared to non-malignant LO2 hepatocytes, while remaining unchanged in HCC-97L and HCC-97H cells (**Figures 1H, I**). Together, these data support that RBP7 is upregulated in HCC and displays a significant relationship with HCC progression.

**Table 1:** The relationship between *RBP7* expression and clinicopathological parameters of HCC patients.

Clinicopathological parameters	Variables	*RBP7* expression		χ^2^	*P* value
		Low	High		
Gender				0.806	0.369
	Female	2	1		
	Male	14	21		
Age (years)				4.498	0.034
	<50	6	2		
	≥50	10	20		
HBsAg				1.666	0.197
	Positive	12	12		
	Negative	4	10		
Liver cirrhosis				3.643	0.065
	Yes	15	15		
	No	1	7		
Tumor number				0.182	0.67
	Single	13	19		
	Multiple	3	3		
Tumor size				0.031	0.861
	<5 cm	9	13		
	≥5 cm	7	9		
Tumor differentiation				6.532	0.038
	Well	0	7		
	Moderate	9	7		
	Poor	7	8		

### *RBP7* and its co-expressed genes regulate critical oncogenic processes in HCC

**Figure 2:**
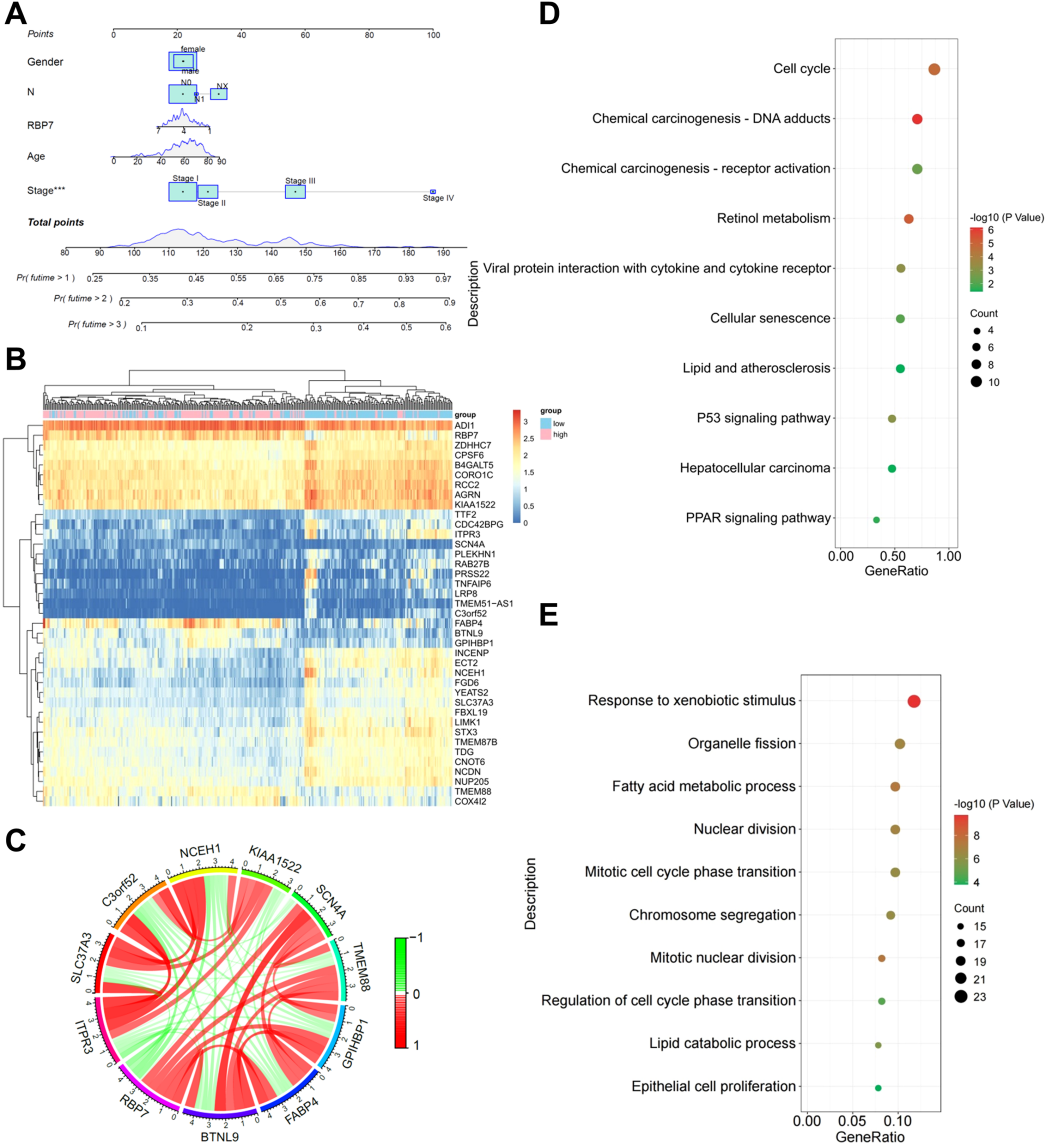
Bioinformatics analysis of the role of *RBP7* and its co-expressed genes in HCC tumorigenesis. **(A)** A predictive nomogram illustrating the relationship between different parameters with OS in HCC patients. **(B)** Heatmap showing the distribution of RBP7 co-expressed genes. **(C)** Correlation circle plot showing the relationship between RBP7 and its co-expressed genes. KEGG **(D)** or GO **(E)** analysis of the major pathways and processes regulated by RBP7 and its co-expressed genes. ***P< 0.001.

We developed a prognostic nomogram to predict OS in HCC patients based on RBP7 expression, gender, N stage, age, and disease stage. The results revealed stage is the most significant prognostic factor (**Figure 2A**). Through comprehensive co-expression network analysis, we identified 649 genes significantly associated with RBP7 expression (**Figure 2B**). Notably, *RBP7* positively correlates with a series of genes, including *BTNL9*, *COX4I2*, *FABP4*, *GPIHBP1* and *TMEM88*, while negatively relates with several genes such as *ECM1*, *GDF2*, *CHST4*, *CLEC1B* and *DSG2* (**Figure 2C**). To further explore the potential biological behaviors of *RBP7* and its co-expressed genes, KEGG analysis was performed and the results showed that these genes are primarily enrich in biological pathways related to cell cycle, drug adducts, retinol metabolism, viral protein interaction with cytokine and cytokine receptor, p53 signaling pathway and PPAR signaling pathway etc. (**Figure 2D**). Gene ontology (GO) analysis further demonstrated involvement in various cellular processes, such as response to xenobiotic stimulus, fatty acid metabolic process, nuclear division, chromosome segregation, cell cycle transition, and epithelial cell proliferation (**Figure 2E**). These data imply that RBP7 plays a role in regulating multiple cellular processes critical for liver function and HCC tumorigenesis.

### RBP7 knockdown strikingly inhibits HCC cell proliferation *in vitro* and *in vivo*

**Figure 3:**
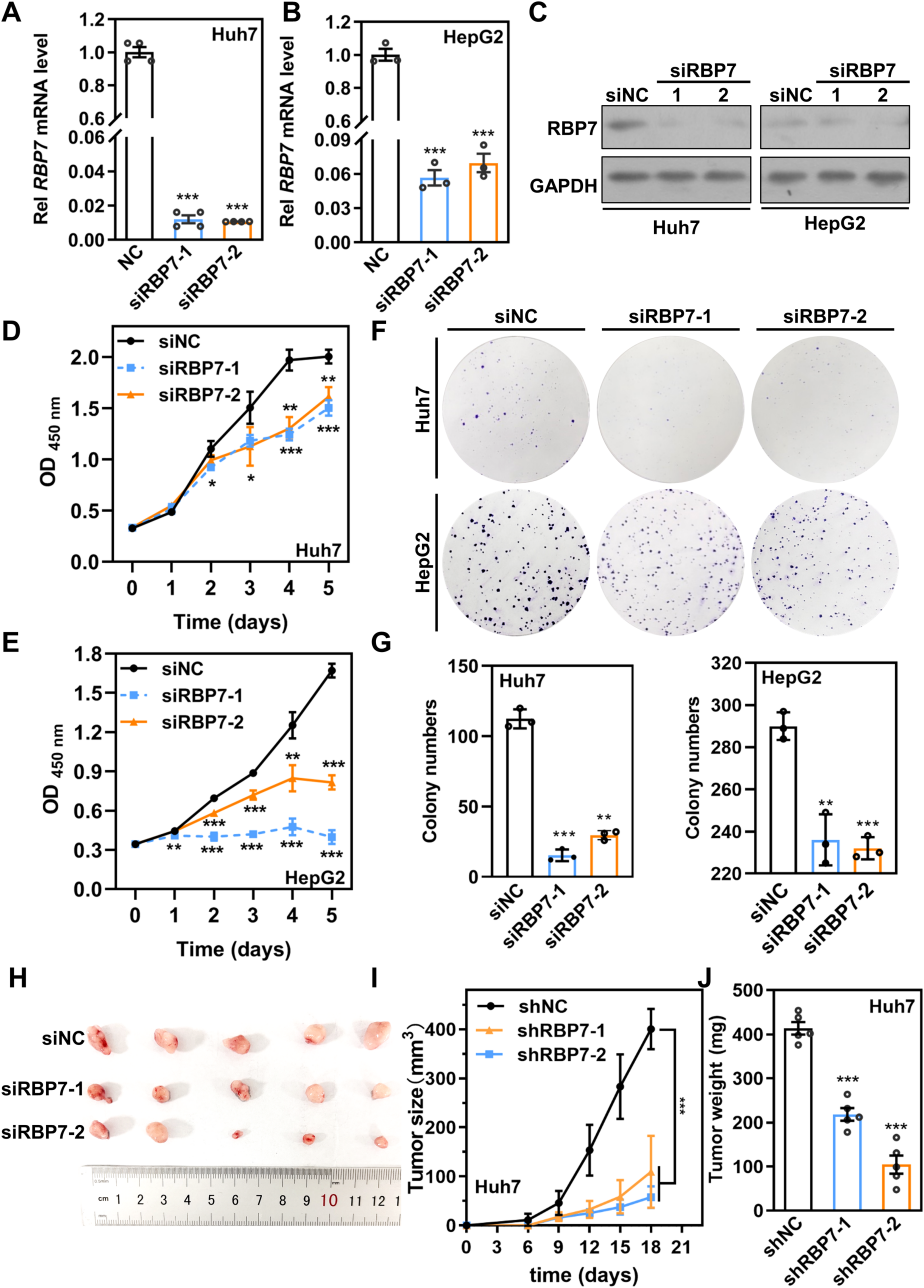
RBP7 KD impairs cell proliferation capacity of HCC cells *in vitro* and *in vivo*. qRT-PCR **(A, B)** or western blot **(C)** results showing the expression of RBP7 in Huh7 and HCC-97H cells after transfection with indicated siRNA. CCK8 assay evaluating the proliferation capacity of Huh7 **(D)** or HCC-97H **(E)** cells after RBP7 KD. **(F, G)** Colony formation assay to assess the cell colony formation ability of HCC cells after RBP7 KD. Colony numbers were counted by Image J software and relative colony number were calculated. Image **(H)**, Tumor growth curve **(I)** and weight of tumors **(J)** of subcutaneous xenograft tumor formed by Huh7 cells with stably RBP7 KD or controls in nude mice (n=5). All experiments, except the xenograft tumor assay, were performed at least three times. Data are shown as mean ± standard deviations. *P* values were determined by Student’s *t* test; *P< 0.05, **P< 0.01 and ***P< 0.001.

Given the strong correlation between RBP7 expression and HCC progression, particularly its involvement in proliferation-related progresses (**Figures 2D, E**), we sought to examine the functional role of RBP7 in HCC cells. To this end, we knockdown (KD) RBP7 expression in Huh7 and HepG2 cells using two independent siRNAs (siRBP7–1 and siRBP7-2). qRT-PCR and western blot results confirmed that efficient RBP7 depletion at both mRNA and protein levels in both cell lines (**Figures 3A–C**). CCK8 results revealed that RBP7 KD markedly impaired the proliferation in both cell lines (**Figures 3D, E**). Furthermore, colony formation assays demonstrated that RBP7 depletion notably suppressed the clonogenic capacity of HCC cells (**Figures 3F, G**). To extend these findings *in vivo*, we constructed stable RBP7-KD Huh7 cells using a lentiviral shRNA system (**Supplementary Figure S2**), and subcutaneously implanted them into nude mice. Consistent with the *in vitro* results, RBP7 KD substantially inhibited tumor growth (**Figures 3H–J**). Collectively, these data indicate that RBP7 is critical for HCC cells proliferation, as its depletion significantly hampers the proliferation both *in vitro* and *in vivo*.

### RBP7 knockdown disrupts cell cycle progression and induces G1 arrest in HCC cells

**Figure 4:**
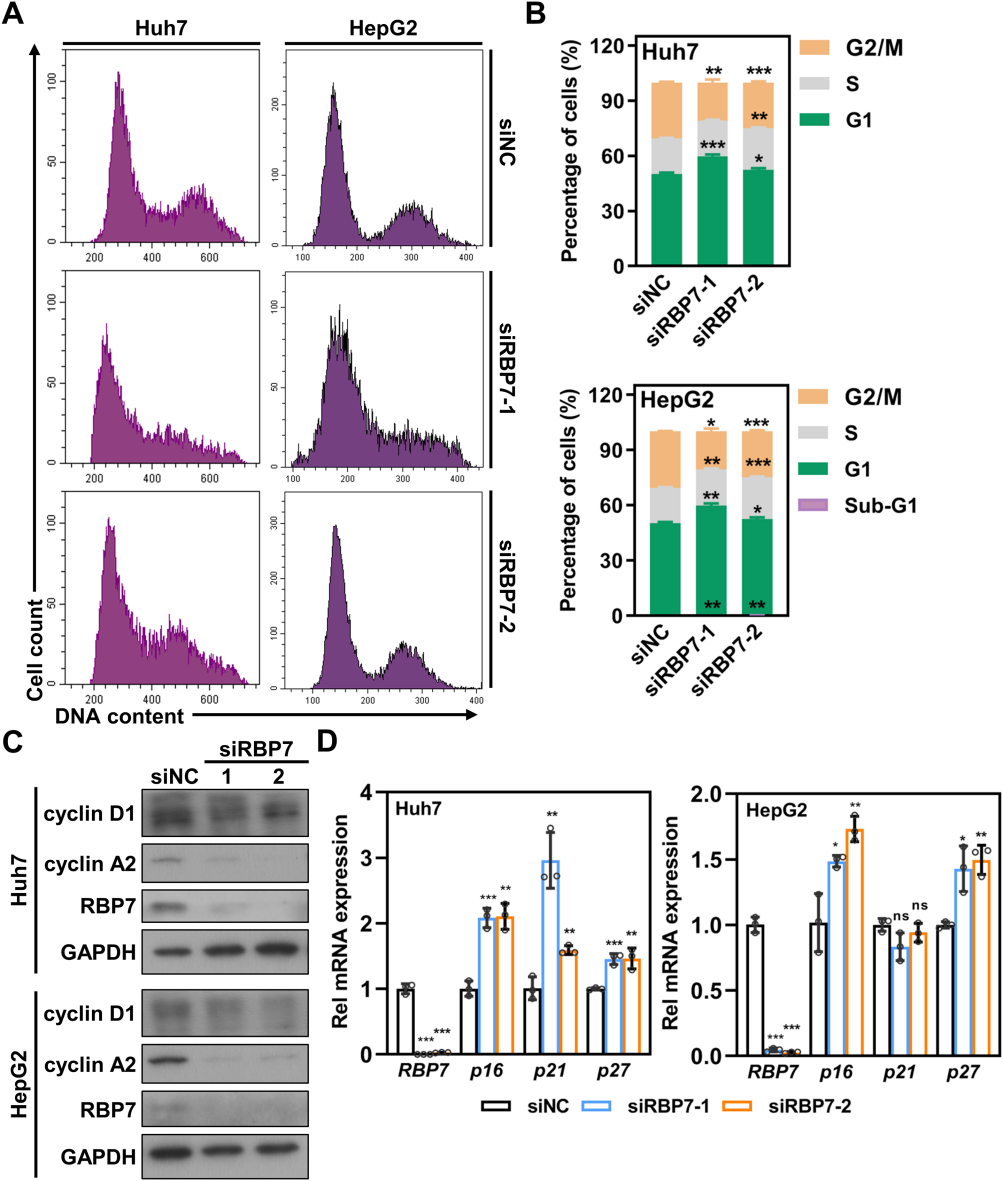
RBP7 KD induces an increase of HCC cells in G1 phase but a reduction in G2/M phase. **(A)** Cell cycle profile of HCC cells after transfection with indicated siRNA. **(B)** Cell cycle distribution based on data shown in **(A)**. **(C)** Western blot results showing the effects of RBP7 KD on the expression of cell cycle related proteins. **(D)** qRT-PCR analysis of the effects of RBP7 KD on the expression of cell cycle inhibitors. All experiments were performed at least three times. Data are shown as mean ± standard deviations. *P* values were calculated using Student’s *t* test; *P< 0.05, **P< 0.01 and ***P< 0.001.

Aberrant cell cycle regulation and uncontrolled cell proliferation are common hallmark features of tumor cells (23). To explore the mechanism underlying RBP7-mediated proliferation in HCC cells, we analyzed the effects of RBP7 on cell cycle progression. Huh7 and HepG2 cells were transfected with either siNC or siRBP7s for 3 days, followed by flow cytometry analysis. The results revealed that RBP7 KD induced a significant accumulation of cells in G1 phase, accompanied by a corresponding decrease in G2/M population (**Figures 4A, B**). Correspondingly, western blot analysis confirmed that RBP7 KD substantially reduced the protein expression of key cell cycle regulators, including cyclin A2 and cyclin D1 (**Figure 4C**). qRT-PCR analysis further demonstrated that RBP7 KD resulted in a significant upregulation of CDK inhibitors *p16* and *p27* (**Figure 4D**). These findings indicate that RBP7 KD impedes cell cycle progression and induces G1 arrest in HCC cells.

### Suppression of RBP7 results in apoptosis in HCC cells

**Figure 5:**
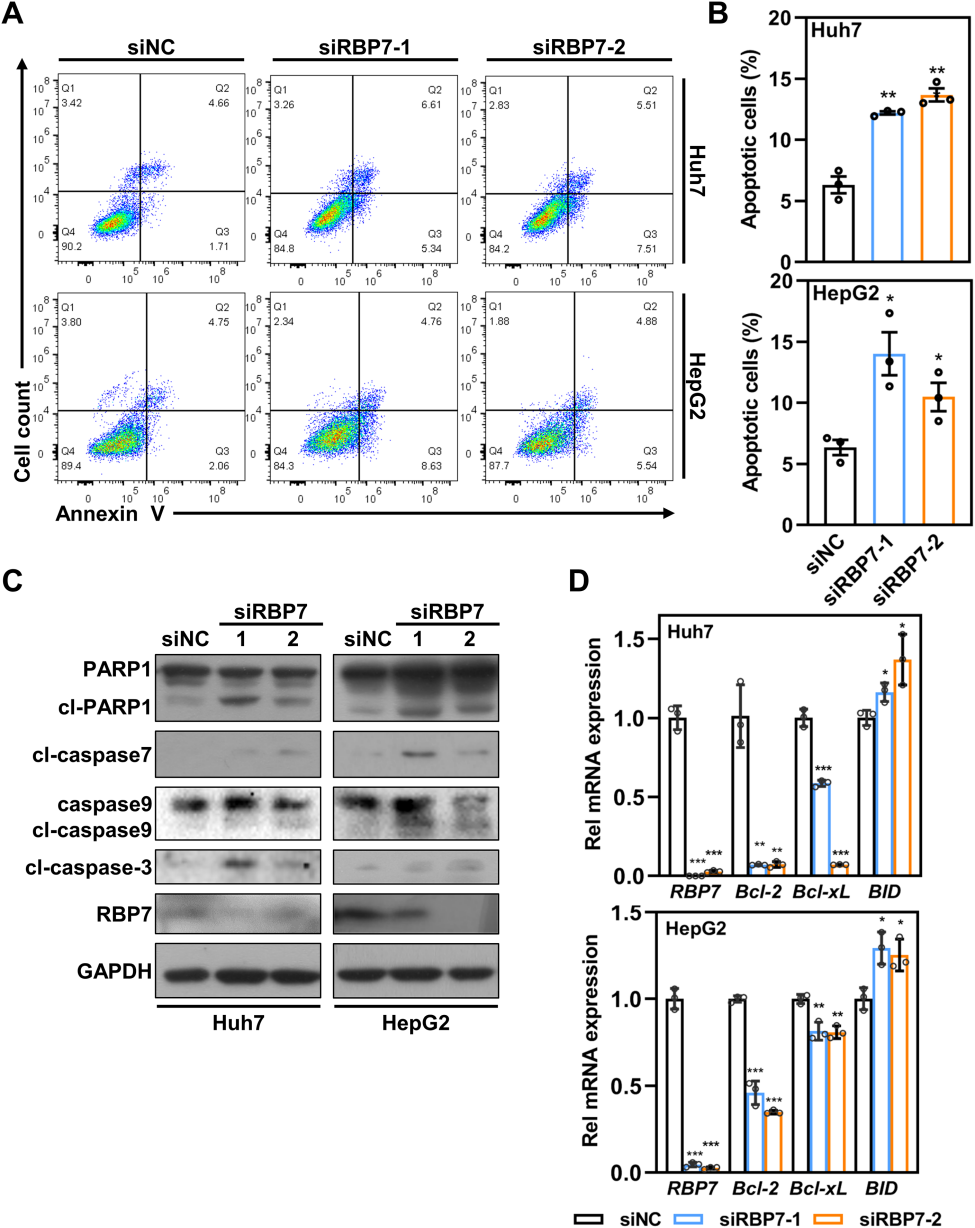
RBP7 KD promotes apoptosis in HCC cells. **(A)** Annexin V/7-AAD staining to analyze cell apoptosis in HCC cells transfected with indicated siRNA. **(B)** Quantification of cell apoptosis based on data from **(A)**. **(C)** Western blot analysis showing the effects of RBP7 KD on the expression of apoptosis-related proteins. **(D)** qRT-PCR showing the effects of RBP7 KD on the expression of apoptosis-related Bcl-2 family genes. All experiments were performed at least three times. Data are shown as mean ± standard deviations. *P* values were calculated using Student’s *t* test; *P< 0.05, **P< 0.01 and ***P< 0.001.

Besides cell cycle arrest, flow cytometry analysis revealed a distinct sub-G1 population in RBP7- KD cells (**Figures 4A, B**), suggesting potential induction of apoptosis. To test this, we performed Annexin V staining and found observed a significant increase in apoptotic cells upon RBP7 depletion in both Huh7 and HepG2 cells (**Figures 5A, B**). Western blot results showing that RBP7 KD strikingly increased the level of cleaved caspase 3, caspase 7 and PARP1 (**Figure 5C**).

Given previous reports that retinal and its analogues can modulate mitochondrial biogenesis and function as toxicants (24, 25), we assessed whether mitochondria-dependent apoptotic pathway is involved in RBP7 KD-induced cell apoptosis. Indeed, RBP7 KD caused a significant increase in cleaved caspase 9 (**Figure 5C**). Consistently, RBP7 KD also induced an increased transcription of pro-apoptotic BH3-only protein *BID* and decreased transcription of anti-apoptotic *Bcl-2* and *Bcl-XL* (**Figure 5D**). Taken together, these findings demonstrate that RBP7 KD induces a mitochondria-dependent apoptosis in HCC cells.

### RBP7 KD suppresses HCC cells proliferation via activating p38 MAPK pathway

**Figure 6:**
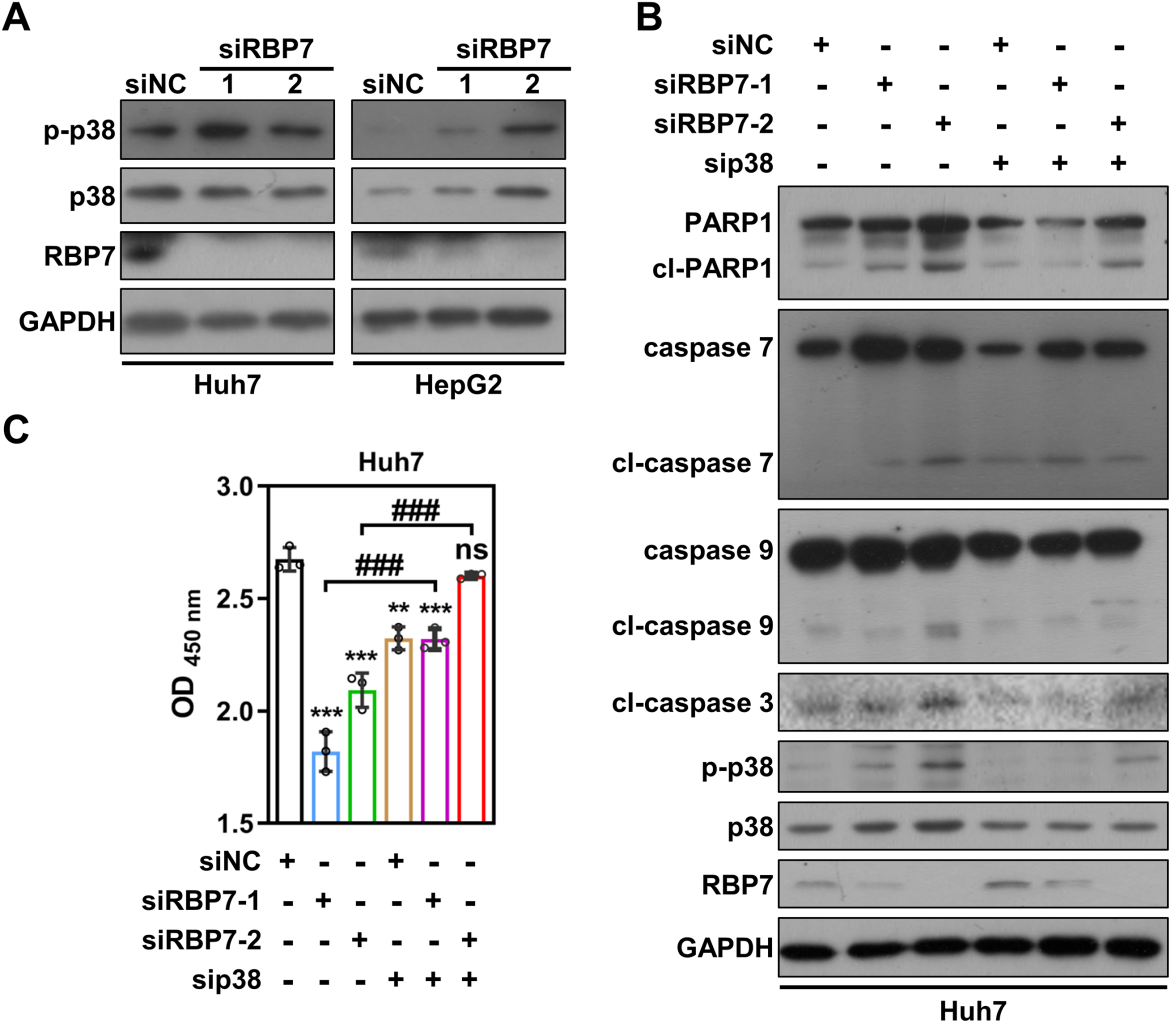
RBP7 KD-induced cell proliferation inhibition depends on p38 MAPK pathway activation. **(A)** Western blot analysis showing the effects of RBP7 KD on p38 MAPK pathway in HCC cells. **(B)** Western blot result showing the effect of p38 siRNA transfection on RBP7 KD-induced changes in apoptosis-related proteins in Huh7 cells. **(C)** Cell count to analyze the effect of p38 siRNA transfection on RBP7 KD-induced cell proliferation inhibition in Huh7 cells. All experiments were performed at least three times. Data are shown as mean ± standard deviations. *P* values were calculated using Student’s *t* test; **P< 0.01 and ***P< 0.001 compare to siNC; ^###^ *P*< 0.001.

To explore the molecular mechanism(s) by which RBP7 regulates HCC cell proliferation, we examined several key proliferation-associated signaling pathways. Among them, we found that p38 phosphorylation was significantly elevated following RBP7 KD in both Huh7 and HepG2 cells (**Figure 6A**). p38 MAPK pathway is known to play a critical role in regulating cell response to extra- and intracellular stress (26). To determine whether the pathway is involved in RBP7 KD-induced cell proliferation inhibition, we used a specific p38 siRNA to reverse the p38 MAPK activation induced by RBP7 KD in HCC cells (**Figure 6B**). CCK8 assay revealed that suppression of p38 MAPK pathway could significantly restore the proliferative capability of RBP7 KD Huh7 cells (**Figure 6C**). Consistently, p38 MAPK inhibition also significantly reduced the caspase activation and PARP1 cleavage triggered by RBP7 KD (**Figure 6B**). Collectively, these finding suggest that p38 MAPK pathway, at least to a large extent, mediates the proliferation inhibition and apoptosis upon RBP7 KD-induced stress in HCC cells.

### RBP7 serves as a potential prognostic biomarker for patients with HCC

**Figure 7:**
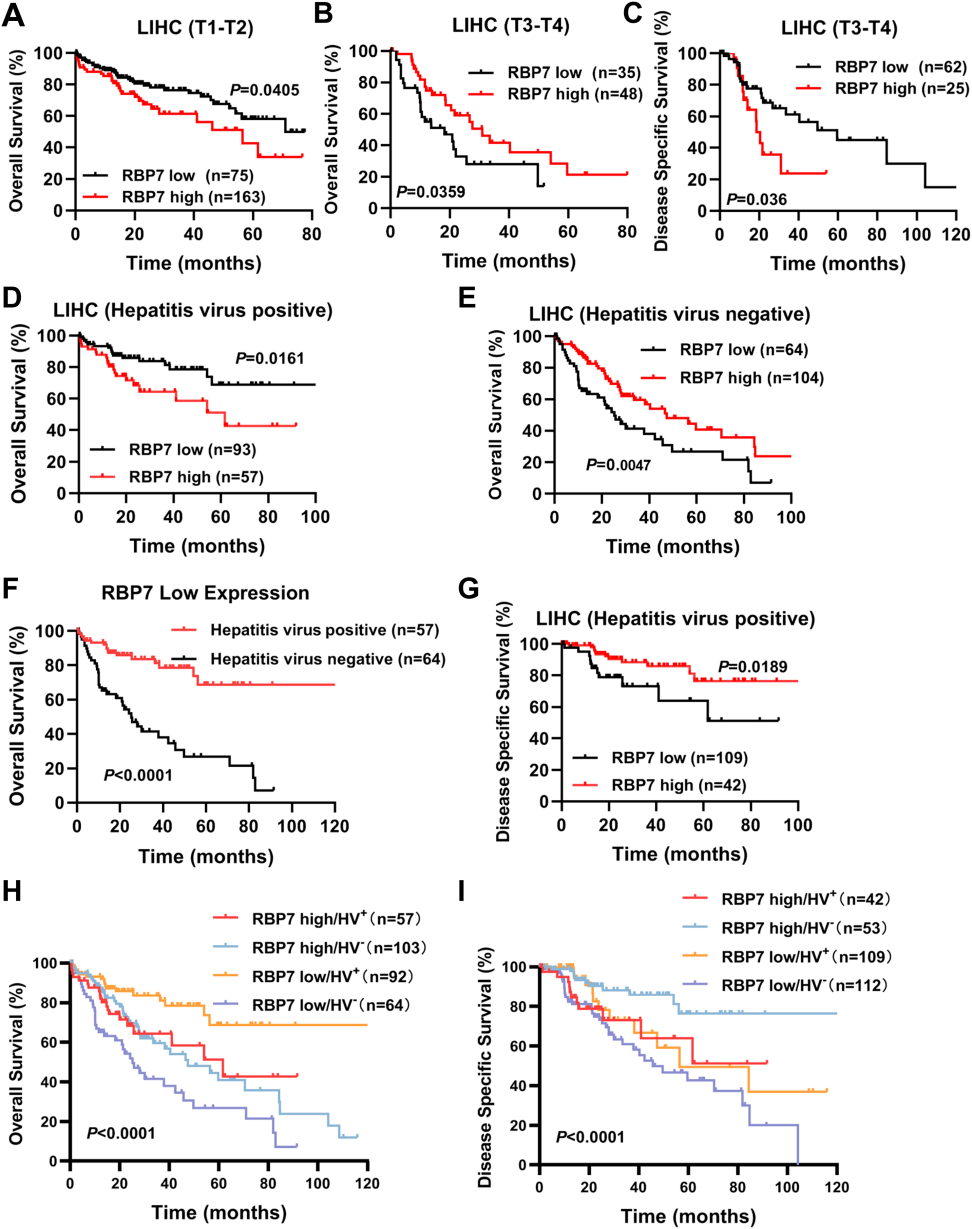
RBP7 closely correlates with prognosis in patients with different subtypes of HCC. TCGA cohort analysis showing the relationship between RBP7 expression and OS in early-stage (T1-T2) **(A)** and late-stage (T3-T4) **(B)** HCC patients. **(C)** TCGA cohort analysis showing the relationship between RBP7 expression and DSS in late-stage of HCC patients. TCGA cohort analysis showing the relationship between RBP7 expression and OS in HCC patient with **(D)** or without **(E)** hepatitis virus infection. **(F)** Survivorship curve illustrating the relationship between hepatitis virus infection and OS in lower RBP7-expressed HCC patients. **(G)** TCGA cohort analysis showing the relationship between RBP7 expression and DSS in hepatitis virus-positive HCC patients. Bivariate analysis showing the combination RBP7 and hepatitis virus infection to predict OS **(H)** or DDS **(I)** in HCC patients from TCGA cohort. Log-rank test was used for survival analysis.

Given the pivotal role of RBP7 in HCC tumorigenesis, we further analyzed its correlation with the clinical outcomes in HCC patients using Kaplan-Meier analysis and log-rank test (https://kmplot.com/analysis/). We found that, although *RBP7* mRNA expression was not significant correlated with OS in the entire HCC patients cohort, it exhibited stage-dependent prognostic value. Specifically, elevated RBP7 expression was associated with poorer OS in early-stage (T1–T2) HCC patients, but with improved OS in those with late-stage (T3–T4) disease (**Figures 7A, B**; **Supplementary Figure S3A**). Additionally, high *RBP7* expression was linked to worse disease-specific survival (DSS) in late-stage HCC patients, whereas no significant association was observed between RBP7 levels and DSS in early-stage cases (**Figure 7C**; **Supplementary Figure S3B**). These findings suggest that RBP7 may serve as a stage-specific prognostic biomarker in HCC.

We further analyzed the prognostic significance of several risk factors including RBP7 using multivariate Cox proportional hazards modeling. The results revealed that T stage ia an independent prognostic factors for poor survival of HCC patients (**Table 2**). Additionally, since a higher *RBP7* mRNA expression was observed in hepatitis virus-positive HCC patients (**Figure 1E**), we next examined whether *RBP7* expression is associated with prognosis of these patients. Our analysis revealed that higher *RBP7* expression was significantly correlated with worse OS in hepatitis virus-positive HCC patients, but with improved OS in hepatitis virus-negative individuals (**Figures 7D, E**). Notably, among patients with low RBP7 expression, those who were hepatitis virus-positive had better OS compared to virus-negative counterparts (**Figure 7F**). However, hepatitis virus infection status did not significantly affect OS among patients with high RBP7 expression (**Supplementary Figure S3C**). Regarding disease-specific survival (DSS), higher RBP7 expression was predictive of better outcomes in hepatitis virus-positive patients, but not in virus-negative ones (**Figure 7G**; **Supplementary Figure S3D**). Bivariate analysis further demonstrated that hepatitis virus-positive patients with low RBP7 expression had the most favorable OS, whereas those with high RBP7 expression still showed better DSS compared to other subgroups (**Figures 7H, I**). Taken together, these findings suggest that RBP7 may serve as a context-dependent prognostic biomarker, particularly relevant to hepatitis virus-associated HCC subtypes.

**Table 2:** the HR (95% CI) and *P* value of different clinicopathological parameters by univariate Cox proportional hazards analysis.

Clinicopathological parameters	HR (95% CI)	*P* value
Gender (Male vs Female)	0.84 (0.58-1.22)	0.359
Age (years) (<65 vs ≥65)	1.13 (0.79-1.63)	0.508
T stage (T3-T4 vs T1-T2)	2.67 (1.84-3.86)	<0.001
Lymph node metastasis (Positive vs Negative)	1.26 (0.75-2.11)	0.390
RBP7	0.81 (0.56-1.17)	0.257

## Discussion

In this study, we demonstrated that RBP7 is significantly elevated in HCC tissues and its expression correlates with patients prognosis across different HCC subtypes. RBP7 KD induces p38 MAPK activation, leading to cell proliferation suppression via induction of cell cycle arrest and apoptosis.

Multiple studies using cell lines, mice models and clinical data have shown that retinoid signaling is downregulated in chronic liver disease and HCC (6). Meanwhile, several retinal compounds have been demonstrated to directly or indirectly inhibit HCC progression, highlighting their potential as CHEMOPREVENTIVE AGENTS (27). RBP family proteins are essential for the transport, metabolism, and signaling transduction of retinoids, which are implicated in regulation of cell proliferation and tumorigenesis. However, RBP family members exhibit distinct, context-dependent functions across different cancer types. For instance, RBP4 is elevated in colon cancer and associated with poor prognosis, whereas it is reduced in HCC and proposed as a potential diagnostic and prognostic biomarker (28, 29). In our study, we found that RBP7 is overexpressed in HCC patients, particularly those with early-stage disease. Elevated RBP7 was positively associated with poor OS in early-stage HCC patients but inversely correlated with OS in those with late-stage HCC. Combined with the observed role of RBP7 in promoting HCC cell proliferation, these findings suggest that RBP7 may function as an oncogene during early tumorigenesis. However, its role in HCC tumorigenesis may be influenced by the tumor microenvironment, including retinoid availability and signaling. Further studies are warranted to elucidate the precise mechanisms underlying the context-dependent functions of RBP7 in HCC and assess its translational relevance in patient samples.

p38 MAPK pathway has been wildly reported to play important roles in regulating cell proliferation, particularly in response to various extra- or intracellular stimuli (30). Several studies have demonstrated that p38 MAPK pathway is also affected by retinoids treatment and mediates cellular response. For instance, Yazan Alsayed et al. found that all-trans-retinoic acid (ATRA) treatment activated p38 MAPK pathway, which exhibited a negative regulatory role in RA-mediated differentiation and proliferation inhibition in acute promyelocytic leukemia cells (31). In contrast, ATRA has been shown to suppress p38 MAPK pathway, leading to decreased proliferation and migration in breast cancer cells (32). These findings highlight the context-dependent nature of p38 MAPK signaling in response to retinoids. Furthermore, Hallahan et al. demonstrated that retinoid treatment promoted increased binding of RAR to the bone morphogenetic protein 2 (BMP2) promoter, subsequently activating p38 MAPK via BMP2 upregulation (33). In our study, we found that RBP7 KD activated p38 MAPK pathway, leading to suppressed cell proliferation and enhanced apoptosis. Given that RBP7 is involved in retinoids transport and metabolism, its depletion may alter intracellular retinoid levels, thereby regulating downstream signaling pathways such as p38 MAPK. Additionally, RBP7 has been reported to mediate the antioxidant properties of PPARγ in endothelium (9, 10), suggesting a potential role in redox homeostasis. Therefore, it remains to be elucidated whether p38 MAPK pathway activation following RBP7 KD is mediated by disrupted retinoid metabolism, alterations in other retinoic acid signaling pathway components, or reactive oxygen species (ROS) accumulation.

Chronic hepatitis virus infection is acknowledged as one of the major risk factors for HCC, contributing to HCC tumorigenesis through repeated cycles of liver injury damage, regeneration, and fibrosis (34, 35). Retinoids and their metabolic pathways have been wildly demonstrated to interact with hepatitis virus infection. For instance, treatment with acyclic retinoid Peretinoin could effectively reduce hepatitis C virus (HCV) release and displayed potent antiviral effect (36). Additionally, silencing of retinoid X receptor alpha (RXRα) has been shown to enhance HBV infection at early stages (37). In turn, the hepatitis C virus core protein can directly interact with RXRα, increasing its DNA-binding activity and upregulating the expression of downstream target genes, including PPARα and RBP2 (38). In this study, we observed that RBP7 is upregulated in hepatitis virus-positive HCC patients. We hypothesize that this elevation may be driven by virus-induced alterations in retinoid metabolism and signaling. These findings suggest a potential link between viral infection and RBP7 regulation, warranting further investigation into the mechanistic crosstalk between hepatitis virus infection and retinoid-related pathways in HCC.

## Conclusion

Our finding showed that RBP7 is frequently enhanced in HCC tumor tissues, and its expression is closely correlated with patient prognosis across different HCC subtypes. Targeting RBP7 inhibits HCC cells proliferation via activation of p38 MAPK pathway, supporting that RBP7 could serve as a promising prognostic biomarker and potential therapeutic target in HCC.

## Data Availability

The original contributions presented in the study are included in the article/**Supplementary Material**. Further inquiries can be directed to the corresponding authors.
