# Supplementary material for: RBP7 knockdown inhibits proliferation of human hepatocellular carcinoma and activates the p38 MAPK pathway
Source: Front Oncol. 2025 Jun 25;15:1592616. doi: 10.3389/fonc.2025.1592616 (PMC12237887; doi:10.3389/fonc.2025.1592616)
Supplement: Supplementary file 1 [file DataSheet1.docx]

**Supplemental figure legend**

**
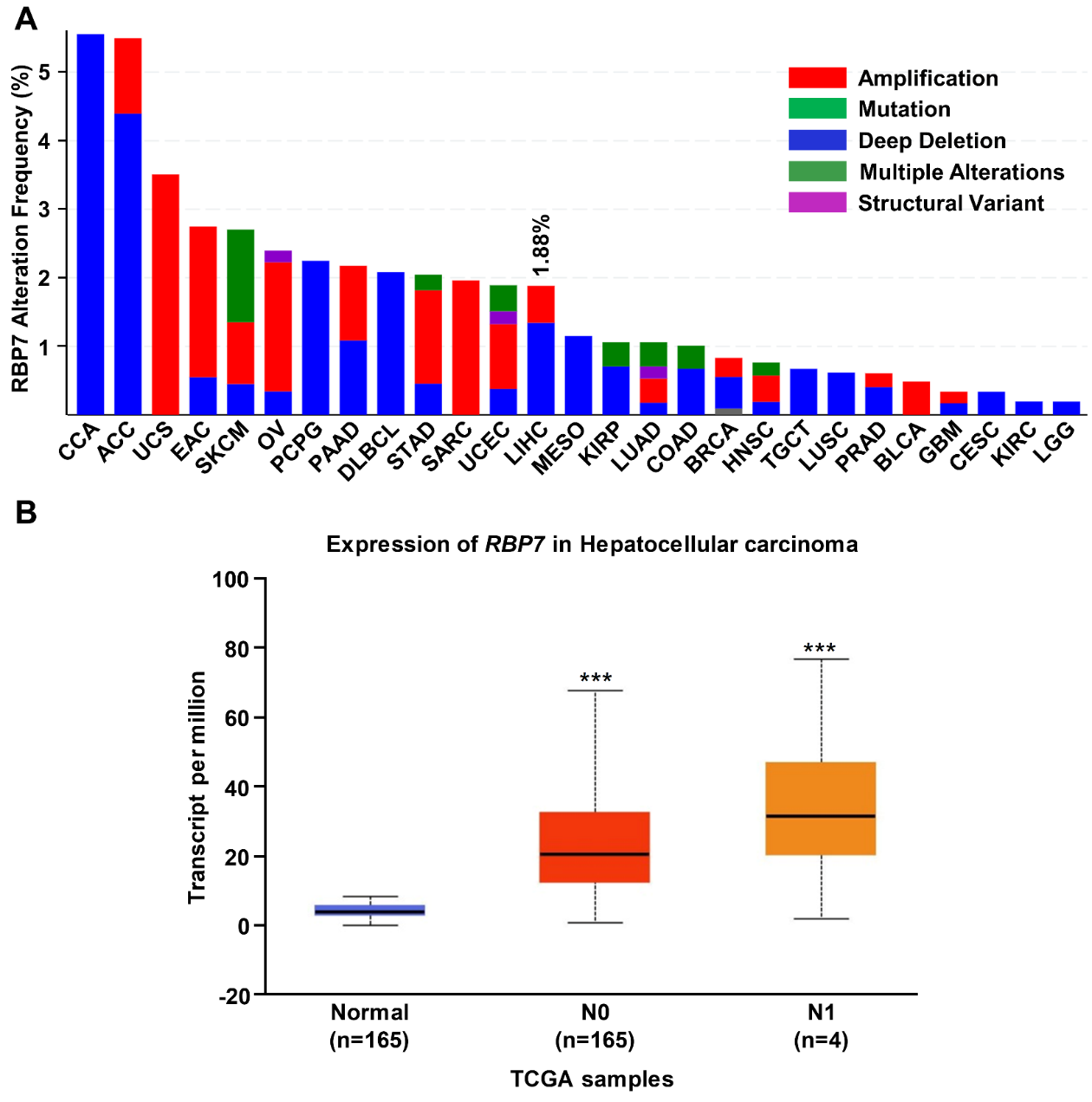
Fig. S1 The variation of *RBP7* in different types of cancer.** (a) Analysis of the variation frequency of *RBP7* in different types of cancer using TCGA data. (b) Analysis of *RBP7* mRNA expression in different stages of HCC patients using TCGA data. ****P*< 0.001**.**


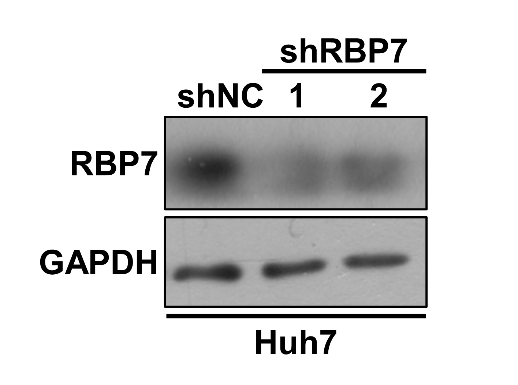


**Fig. S2 Western blot analyze the expression of RBP7 in Huh7 cells with stable RBP7 KD.**

**
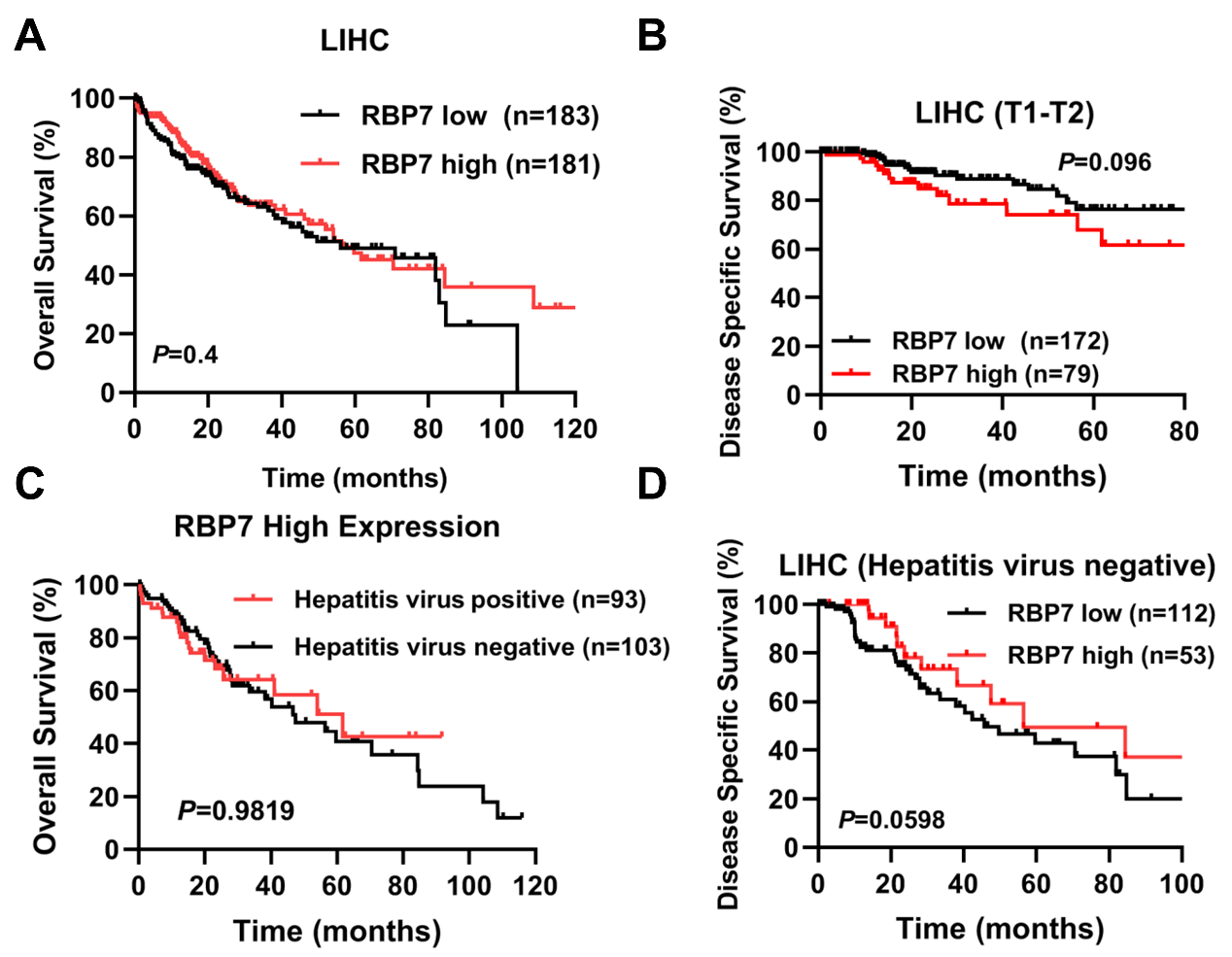
**

**Fig. S3 The relationship between RBP7 and prognosis in patients with different subtypes of HCC.** TCGA cohort analysis showing the relationship between RBP7 expression and OS in HCC patients. (B) TCGA cohort analysis showing the relationship between hepatitis virus infection and OS in RBP7 high expressed HCC patients. (D) TCGA cohort analysis showing the relationship between RBP7 expression and DSS in HCC patient without hepatitis virus infection. Log-rank test was used for survival analysis.
